# MassArray Genotyping as a Selection Tool for Extending the Shelf-Life of Fresh Gilthead Sea Bream and European Seabass

**DOI:** 10.3390/ani14020205

**Published:** 2024-01-08

**Authors:** Rafael Angelakopoulos, Andreas Tsipourlianos, Themistoklis Giannoulis, Zissis Mamuris, Katerina A. Moutou

**Affiliations:** 1 Laboratory of Genetics, Comparative and Evolutionary Biology, Department of Biochemistry and Biotechnology, School of Medical Sciences, University of Thessaly, Viopolis, Mezourlo, 41500 Larissa, Greece; rangelak@uth.gr (R.A.); a.tsipourlianos@gmail.com (A.T.); zmamur@uth.gr (Z.M.); 2 Laboratory of Biology, Genetics and Bioinformatics, Department of Animal Science, University of Thessaly, Greece Gaiopolis, 41334 Larissa, Greece; thgianno@uth.gr

**Keywords:** muscle deterioration, shelf-life of fresh fish, genetic polymorphism, *Sparus aurata*, *Dicentrarchus labrax*, fillet quality, proteolytic enzymes

## Abstract

**Simple Summary:**

This study focused on improving the fillet quality of European seabass and gilthead sea bream in aquaculture by exploring the genetic basis of fillet degradation after harvest. We identified specific SNPs related to enzymes affecting fillet quality and associated them with enzymatic activity using genotyping. By integrating this platform into breeding programs, we could enhance the shelf-life of fish products in a cost-effective manner. This is crucial for addressing the challenge of fresh fish perishability, ultimately reducing food waste and production costs in the aquaculture industry.

**Abstract:**

In modern aquaculture, genomics-driven breeding programs have emerged as powerful tools for optimizing fish quality. This study focused on two emblematic Mediterranean fish species, the European seabass (*Dicentrarchus labrax*) and the gilthead sea bream (*Sparus aurata*), with a primary aim of exploring the genetic basis of white muscle/fillet degradation in fresh fish following harvest. We identified 57 and 44 missense SNPs in gilthead sea bream and European seabass, respectively, located within genes encoding for endogenous proteases responsible for fillet quality. These SNPs were cherry-picked based on their strategic location within the catalytic/regulatory domains of endogenous proteases that are expressed in the white muscle. Using MassArray technology, we successfully associated differentiated enzymatic activity of those endogenous proteases post-harvest as a phenotypic trait with genetic polymorphism of six SNPs in gilthead sea bream and nine in European seabass. These findings can be valuable attributes in selective breeding programs toward the extension of freshness and shelf life of these species. The integration of MassArray technology into breeding programs offers a cost-effective strategy for harnessing the potential of these genetic variants to enhance the overall quality of the final product. Recognizing that fresh fish perishability is a challenge, extending shelf-life is pivotal in reducing losses and production costs.

## 1. Introduction

One of the main pursuits in modern aquaculture is to increase the shelf life of the fresh final product, thus minimizing losses and overall production costs. Seafood is extremely perishable and typically degrades faster than other types of muscle foods. The extent to which these changes occur over time dictates the product’s shelf life [1]. Fish are more susceptible to textural deterioration post-mortem because of biochemical and microbiological deterioration due to their high moisture content, reactive endogenous enzymes, and enhanced nutrients [2]. As a result, significant spoilage of fish occurs at various points along the production chain (post-harvest handling, processing, storage, and distribution), with considerable economic losses, product quality degradation, and customer safety concerns [3]. Biochemical changes have a significant effect on the deterioration of the quality of fish fillets. These changes can be metabolic or structural (e.g., changes in the myofibrillar and changes in the extracellular matrix), all of which are triggered by endogenous proteases [4,5,6]. Proteases that contribute to myotomia degradation can originate from both muscle tissue and the digestive system, provided the latter has not been removed prior to storage [7]. Collagenases [8,9], which hydrolyze connective tissue collagen, as well as cathepsins [5,10] and calpains [11,12], which proteolyze muscle fibril proteins, play a critical part in this process.

These enzymes belong to multi-member gene families, with a plethora of members being expressed in the white muscle tissue of European seabass and gilthead sea bream. The genetic variability in these proteolytic enzymes can be used as a tool for genomic selection and prolongation of fillet shelf life [13]. Shelf life is the period before a food product is considered unsuitable for consumption or sale. During the last several years, reliable methods have been developed to extend the shelf life of food products with formulation, processing, or packaging innovations [14,15,16,17,18,19].

European seabass and gilthead sea bream are the two emblematic fish species in Mediterranean marine aquaculture. At the European level, they rank third and fourth, respectively, in value after Atlantic salmon and trout [20]. Modern fish farming has embraced the importance of genetic selection using existing genomic technologies to estimate well-characterized genetic diversity and enhance broodstock formation and selection approaches [21]. Achieving the goal of genetically selecting and improving a population in the context of breeding programs often necessitates the production of genetic data for whole genomes, such as single-nucleotide polymorphisms (SNPs), from a significant number of individuals. When these polymorphisms are associated with a specific trait, this information can be utilized for targeted parental selection to ensure the prevalence of the desired traits in a population [22]. Over the years, significant genomic tools for European seabass and gilthead sea bream have been developed, including the sequencing and annotation of their whole genomes [23,24]. Over the last decade, genome-wide association studies (GWASs) have contributed significantly to new discoveries of genes related to various traits. Despite the array’s utility for gene identification, a fundamental need remains for platforms that enable the affordable and effective genotyping of a customized SNP list for certain parts of the genome. For instance, once SNPs associated with a particular phenotype are identified in a GWAS analysis, replication of the findings in a second sample is often required. Often, only a few dozen SNPs require genotyping at this time [25]. Consequently, a substantial fraction of the data generated in a GWAS is redundant, resulting in inefficient resource utilization [26]. MassArray technology is an approach that is appropriate for reproducing polymorphisms in a second population. The Agena Bioscience MassARRAY^®^ system is a genotyping platform that enables the genotyping of tens to hundreds of user-defined SNPs in hundreds or thousands of high-performance DNA samples. Multiplex PCR design is accomplished by grouping selected SNPs (up to 40 suitable SNPs) [25,27].

To our knowledge, this is the first attempt to use genotyping to identify polymorphisms associated with this specific trait and generate data that can be utilized for parental selection in Mediterranean-farmed fish species.

The objectives of this study were (i) to identify variants in genes encoding for calpains, cathepsins, and metalloproteases responsible for muscle deterioration in gilthead sea bream (*Sparus aurata*) and European seabass (*Dicentrarchus labrax*); (ii) to genotype missense variants, as they are known to alter the genetic code affecting the function of a protein, and to select those located within crucial domains for the protein function; and (iii) to explore possible associations between the selected variants and the enzymatic activity of the aforementioned proteases.

## 2. Materials and Methods

### 2.1. Ethics Statement

All examined biological materials were derived from fish reared and harvested at commercial farms registered for aquaculture production in EU countries. Animal sampling followed routine procedures, and the samples were collected by a qualified staff member from standard production cycles. The legislation and measures implemented by the commercial producers complied with existing national and EU (Directive 1998/58/EC) legislation (protection of animals kept for farming).

### 2.2. Animal Selection for Whole Genome Sequencing

Whole genome sequencing was performed on both species using Illumina platforms. For the European seabass, DNA from five individuals was mixed equimolarly. For gilthead sea bream, 24 individuals were selected from various European aquaculture farms and were split into four sequencing pools. For the fastq files produced, the quality of the reads was evaluated using FASTQC [28], and low-quality reads (minimum PHRED score: 30), as well as adapter sequences, were discarded with Trimmomatic [29]. Then, the reads were aligned to the reference genomes using the Burrows–Wheeler aligner (BWA) [30]. SAM files were converted into BAM files using SAMtools [31] and finally, variant calling was performed using freeBayes [32]. The variant calling file (VCF) was used to find the alternate variant in contrast with the reference genomes (*Sparus aurata*: GCA_900880675.1, *Dicentrarchus labrax*: http://public-genomes-ngs.molgen.mpg.de/cgi-bin/hgGateway?db=dicLab1, accessed on 4 March 2021). The detailed pipeline used for the analysis can be found on GitHub (https://github.com/RafaelAngelakopoulos/Bioz_lab/tree/0f040a4aee3536952a6df587f25a02ddb74fa61b/WGS, accessed on 12 December 2023).

After annotating the variants mapped in the genes responsible for proteolysis (calpains, collagenases, and cathepsins) a filtering step was performed, selecting missense variants in genes that are expressed in white muscle tissue and preferably those mapped in the catalytic/regulatory domains of the enzymes. Public RNAseq data, *Sparus aurata:* SRR6237499 and *Dicentrarchus labrax:* ERR9715622, were used to identify calpain, collagenase, and cathepsin genes expressed in white muscle.

### 2.3. Animal Selection for Genotyping

Fish were of commercial size (300–500 g) and were sacrificed using approved slaughtering methods. A total of 166 gilthead sea bream and 201 European seabass individuals, reared in two different Greek aquaculture farming units, were selected for DNA and enzymatic extraction.

### 2.4. Enzymatic Phenotyping

On harvest day, the activity of calpain, collagenase, and cathepsin was determined in the gilthead sea bream and European seabass samples. White muscle samples (200 mg) were extracted from the fish fillet and immediately snap-frozen in liquid nitrogen and kept at −80 °C until further investigation, as previously described [4,33]. Briefly, calpain, collagenase, and cathepsin B and L enzymatic activity were assayed using the Barret and Kirschke method, with minor modifications. L-methionine-AMC trifluoroacetic salt in DMSO and Suc-Gly-Pro-Leu-Gly-Pro-AMC in DMSO were used as calpain and collagenase substrates, respectively. Enzyme extracts were thoroughly mixed with an appropriate substrate buffer solution containing 100 mM bis-Tris and 5 mM CaCl_2_ at a pH of 6.5. Cathepsin B and L activity were determined using proper substrates, i.e., Z-arginine-arginine-7-amido-4-methyl-coumarin hydrochloride and Z-phenylalanine-arginine-7-amido-4-methyl-coumarin hydrochloride, respectively. The enzyme extract was mixed with the substrate solution (pH 6.5, 100 mmol/L Tris-HCl, 20 mmol/L EDTA, and 4 mmol/L DTT) [1]. A spectrofluorometer (VarioskanTM LUX multimode microplate reader, Thermofisher, Waltham, MA, USA) was used to measure the fluorescence of 7-amino-4-methylcoumarin (AMC) released from each and every substrate used (excitation = 360 nm, emission = 460 nm). The protein content of the crude extracts was measured in triplicate using the Bradford method with bovine serum albumin as a reference [34]. Fluorescence units (FUs) per minute and mg of protein were used to calculate enzymatic activity. The enzyme activities in each sample were assayed in duplicate.

### 2.5. DNA Extraction

Total DNA was extracted from the white muscle tissue of all individuals, a procedure necessary for genotyping the selected variations, and stored at −20 °C. The PureLink ™ Genomic DNA Mini kit from Invitrogen (Invitrogen, Catalog number: K182002) was used to extract the DNA from the samples according to the manufacturer’s instructions. DNA quality was assessed using agarose gel electrophoresis and quantified with photometric measurement (Quawell, Q3000) at 260 nm. Samples were properly diluted to 50 ng/μL and sent to Inqaba Biotechnical Industries (Pty) Ltd. (Pretoria, South Africa) for primer synthesis (Supplementary Tables S1 and S2) and genotyping using a MassArray system.

### 2.6. Data Filtering and Association Analysis

The genotypic data for the loci of interest (57 SNPs for gilthead sea bream and 44 SNPs for European seabass) were converted into ped format, and a quality control procedure was performed using PLINK 1.9 [35] to generate reliable data and avoid false positive results in the downstream statistical analysis. Therefore, for quality control, we removed SNPs and individuals based on genotypic and individual missingness. Then, we discarded SNPs with a minor allele frequency of less than 5% and checked the Hardy–Weinberg equilibrium to exclude SNPs that deviated significantly from it, and a threshold of 5% was set for the individual missingness.

SNPstats, a tool for the analysis of the association of genetic polymorphisms (SNPs) with a phenotype, developed by the Institut Catala d’ Oncologia (ICO), was used to process the data derived from the genotype [36]. In terms of statistics, the association with the response (enzymatic activity) was modeled using linear regression models in order to evaluate the rate of variation in the response explained by the polymorphisms using multiple inheritance models [36,37]. Tables with allele and genotype frequencies were generated along with tables showing the association between SNPs and the enzymatic activity per inheritance model (Supplementary Tables S3–S17).

### 2.7. SIFT Algorithm for Amino Acid Substitution Prediction

The Sorting Intolerant from Tolerant (SIFT) algorithm was used to estimate the effect of amino acid substitutions on protein function, and the results were integrated with other functional annotations. SIFT generates predictions by evaluating the properties of the amino acids involved in a specific substitution as well as the evolutionary conservation of the affected region in the protein. It starts by aligning the protein sequence of interest with related protein sequences from other species. This alignment is then used to pinpoint evolutionarily conserved regions that are more likely to be functionally important. SIFT then considers the amino acid properties at the specific substitution position, such as size, charge, polarity, and other chemical properties. It utilizes of this knowledge to predict the effect of the substitution on the structure and function of the protein. Finally, the algorithm calculates a SIFT score for the substitution by combining information about the properties of the substituted amino acid with the evolutionary conservation of the affected region. The SIFT score goes from 0 to 1, with lower values suggesting a higher possibility that the mutation would impair protein function [38].

## 3. Results

### Whole Genome Sequencing and Genotyping

Approximately ~80 M reads per sample and 95% of the reads of the whole genome sequencing passed the quality control criteria.

**Figure 1:**
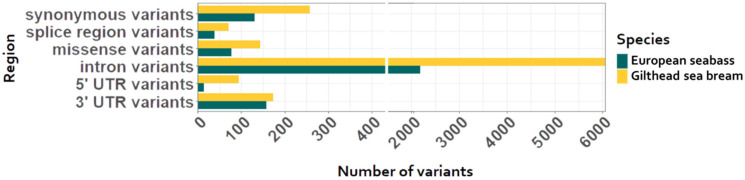
Functional annotation of all variants in the genes of interest in both species, European seabass (*Dicentrarchus labrax*) and gilthead sea bream (*Sparus aurata*).

In total, 6800 and 2608 SNPs for gilthead sea bream and European seabass, respectively, were detected in the genes encoding for calpains, cathepsins, and collagenases and expressed in white muscle (Table 1 and Table 2). More specifically, most variants were found in intronic regions both in European seabass and gilthead sea bream followed by synonymous and untranslated region variants (UTRs). The functional annotation of these SNPs was performed using the SnpEff tool [39] and is presented in Figure 1.

Using PLINK 1.9 and a 5% cutoff for individual missingness, five individuals from the gilthead sea bream dataset and 16 individuals from the European seabass dataset were excluded from the downstream statistical analysis.

**Figure 2:**
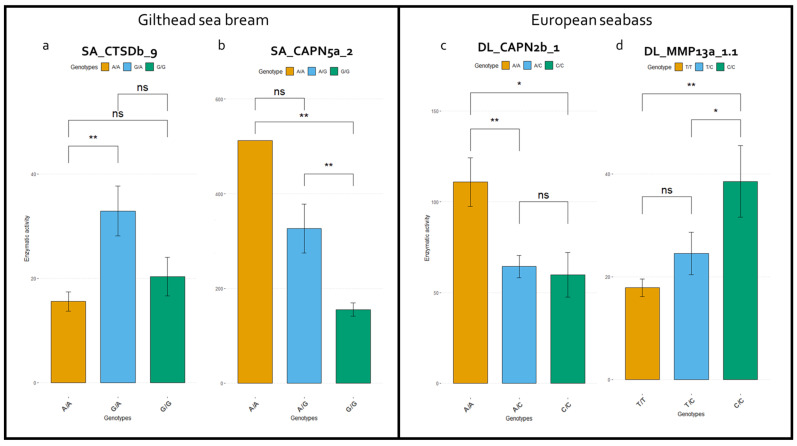
Enzymatic activity per genotype (ns: *p* > 0.05, *: *p* < 0.05, **: *p* < 0.01). Examples of overdominant (**a**), additive (**b**), dominant (**c**), and recessive (**d**) models of inheritance. The two first SNPs are associated with enzymatic activity in gilthead sea bream and the latter (**c**,**d**) in European sea bass.

Among the 57 and 44 SNPs selected for genotyping for gilthead sea bream and European seabass, respectively, 31 and 8 SNPs, were found to be monoallelic or to have failed genotyping. The association analysis revealed several SNPs to be statistically significantly associated with enzymatic activity. Enzymatic activity was calculated for calpain, collagenase, and cathepsins in both species from white muscle samples, as previously described [4]. The allele frequencies of statistically significant variants are reported in Table 3. Table 4 summarizes the changes in enzymatic activity for each variant including the *p*-value for the computed linear regression. Indicative figures regarding the enzymatic activity for each genotype are provided in Figure 2 (two SNPs for each species), and the rest are provided in the Supplementary Materials (Figures S1–S11). Notably, none of the SNPs identified and genotyped are located in the active site of the enzymes, even though several are located within protein domains.

After genotyping the variants, we sought to assess the tolerability of the observed amino acid changes using the Sorting Intolerant from Tolerant (SIFT) algorithm. Our analysis revealed that two mutations in *Sparus aurata* and one mutation in *Dicentrarchus labrax* were non-tolerated. Of note, the mutation in the *capn5a* and *capn10* genes of gilthead sea bream exhibited low frequency in the population, in contrast to the alteration observed in the *MMP13b* gene of European seabass. While our analysis identified two mutations as non-tolerated, it is important to note that some substitutions may have been erroneously predicted to affect function due to the limitation of the SIFT algorithm that considers the diversity of the sequences used (Table 5).

## 4. Discussion

Traditional selection strategies based on phenotypic information were beneficial in boosting the profitability of livestock species in earlier decades. However, these approaches have biological constraints and limitations that are not encountered when using the information in SNPs, which are the primary source of genetic variability across individuals of the same species [40]. Therefore, one of the main aims of genomics analysis is to locate SNPs that impact the functionality and activity of gene products. The identification of associated polymorphisms is critical not only for a better understanding of their genetic basis (i.e., identifying the causal genes) [41,42] but also for the design of genetic selection programs [43].

In this regard, the current study focuses on the relationship between missense SNPs in genes encoding for enzymes driving postmortem degradation of fish white muscle and the actual enzyme activity.

Proteolytic enzymes compromise fish fillet firmness and hardness [44]. The activation of these proteases or their synergistic actions cause autolysis of myofibrils in fish, which results in postmortem muscular weakness [6]. Enzymatic activity determines the severity of the proteolysis, i.e., how rapidly the fillet degrades. As previously noted, all the SNPs used in this investigation are missense variants that alter an amino acid sequence. These alterations are probably involved in changes in protein structure and functionality [45,46].

Calpains are intracellular endopeptidases that initiate myofibril proteolytic breakdown. Four SNPs (SA_capn10_11, SA_capn10_14, DL_capn14b_1, DL_capn5b_3) associated with differential enzymatic activity in both species are located in the CysPc domain of the calpain family (Table 2). The crystal structure of various classical calpains revealed that the core protease domain (CysPC) is composed of two sub-domains containing a catalytic triad [11]. In the presence of Ca^2+^, these two sub-domains are probably reoriented to assemble a cysteine protease active site. Three SNPs (SA_capn5a_1, SA_capn5a_2, DL_capn5b_5) (Table 2) in both species are located in the C2 domain, a calcium and phospholipid binding domain of the *Capn5* gene [47]. This gene belongs to a variation in the non-classical calpains, the TRA-3 group, which contains one C2L domain and one C2 domain in tandem. This domain is important for binding/recognizing substrates and for *calpastatin* binding, which is in contact with the C2 domain [11,48].

*Capn2b* has an SNP in the EF-hand domain. The EF-hand is a Ca^2+^ binding domain with the typical structure of EF-hands [49,50]. Regularly, there are five (5) EF-hand motifs; one of them binds with the regulatory subunit, unifying the heterodimers. The result of this binding is the activation of the enzyme [50]. The *Capn15b* gene is a member of the SOL subfamily. The main structural variations in the SOL subfamily concern the several Zn^2+^-finger motifs, that interact with the target substrate within the N-terminal domain and with a specific SOL-homology domain at the C-terminus of the core protease domain (CysPC) [51]. An SNP in the Zn^2+^-finger motifs was found that can probably affect the interaction with the target substrate.

Cathepsins are lysosomal cysteine proteases that assist in intracellular protein breakdown and turnover [52]. A variant in the peptidase A1 domain has been identified in the *CTSDb* gene. This domain is one of the two monomers composed of two asymmetric lobes (“bilobed”). Each of the lobes provides a catalytic Asp residue, positioned within the hallmark motif Asp-Thr/Ser-Gly, to the active site [53].

In a fish fillet, myotomes are held together by connective tissue called myocommata, which are surrounded by collagenous fibrils [54]. Collagenases are matrix metalloproteases that degrade collagenous fibrils, producing the characteristic gaps found in chilled fish fillets [5,9]. Two SNPs in both *MMP13* paralogs were located in the PGBD domain, which appears to affect MMP enzymatic activity and is located in the region of the gene referred to as the proteoglycan binding domain. As a proteoglycan binding-like domain of MMPs, this domain seems to bind to proteoglycan molecules [55], which are a very important component of the extracellular connective tissue plexus, thus proceeding to the degradation of proteoglycans [56], as well as indirectly participating in the regulation of the concentration of molecules such as chemokines [57]. Another study concluded that the interaction between a pre-MMP and proteoglycans participates in its activation, possibly by bringing it close to some membrane activator [58]. Many different proteoglycans appear to bind to collagen and to differentially regulate the formation and degradation of collagen fibrils, as discussed by [59]. Based on these findings, it is plausible that the proteoglycan-binding domain regulates MMP activation or directs MMPs to approach collagen by binding to collagen-bound proteoglycans, therefore facilitating collagen proteolysis.

We examined the association between nine SNPs in European seabass and six SNPs in gilthead sea bream and their association with enzymatic activity, with the aim of identifying genetic markers for use in breeding programs. Of these SNPs, including three in European seabass and one in gilthead sea bream, the heterozygous genotypes were associated with the preferable phenotype, i.e., a lower enzymatic activity compared with both homozygous genotypes [60]. This phenomenon is likely due to a decrease in enzymatic activity or protein stability in the heterozygous state, resulting in a lower response phenotype [61]. Conversely, the remaining six SNPs in European seabass and two SNPs in gilthead sea bream displayed a dominant/recessive interaction, where one of the homozygous genotypes had a significantly lower response compared with the other homozygous genotype and the heterozygote [62]. For two SNPs in gilthead sea bream, both homozygous genotypes were not present in the population studied, resulting in only the heterozygous genotype and one of the homozygous genotypes being observed (Tables S1–S15).

Finally, we cannot overlook that genes that perform critical functions in the cell are typically under strong evolutionary pressure to avoid accumulating deleterious mutations [63]. This is especially true for enzymes that play crucial roles in regular metabolism, as missense mutations in these genes can have severe consequences for the cell and organism’s survival. Therefore, the fact that some of the SNPs examined in this study displayed a heterozygote advantage may suggest a more complex evolutionary process at play [64,65].

## 5. Conclusions

Among the 57 and 44 SNPs selected, 9 and 6, respectively, for European seabass and gilthead sea bream appeared to be associated with changes in enzyme activity in the population used for the analysis, which is a very modest number compared with those initially selected. We acknowledge that the fish populations studied were of limited size and stress the importance of further investigation to validate our findings.

The 15 non-synonymous polymorphisms found to be associated with the proteolytic activity of these genes, which are actively involved in proteolysis, can be incorporated into genetic improvement programs to select parents exhibiting desired traits (lower proteolytic activity). For the first time, these findings provide the basis for extending parental selection in breeding programs to improve/extend the shelf life of the final product, indicating that low-cost genotyping techniques are of great importance for selecting a specific trait. The combination of the variants arising from the current study can be used to extend the freshness and shelf-life of these emblematic Mediterranean fish.

## Figures and Tables

**Table 1:** Genes expressed in the white muscle of gilthead sea bream. High: gene expressed in white muscle (logreads > 5), Low: low expression of gene in white muscle (logreads < 5), No: gene not expressed in white muscle.

Gene ID	Gene Name	Expression	Gene ID	Gene Name	Expression
ENSSAUG00010000077	*capn11a*	Low	ENSSAUG00010005776	*CTSAa*	Low
ENSSAUG00010008141	*capn11b*	No	ENSSAUG00010008071	*CTSBa*	High
ENSSAUG00010025995	*capn11c*	High	ENSSAUG00010003083	*CTSBb*	Low
ENSSAUG00010003429	*capn14a*	High	ENSSAUG00010007964	*CTSC*	High
ENSSAUG00010016749	*capn14b*	Low	ENSSAUG00010015701	*CTSDa*	High
ENSSAUG00010016757	*capn14c*	Low	ENSSAUG00010016838	*CTSDb*	High
ENSSAUG00010012619	*capn15a*	Low	ENSSAUG00010016344	*CTSDc*	High
ENSSAUG00010016176	*capn15b*	Low	ENSSAUG00010024233	*CTSF*	High
ENSSAUG00010000032	*capn2a*	High	ENSSAUG00010015817	*CTSHa*	No
ENSSAUG00010026026	*capn2b*	Low	ENSSAUG00010021061	*CTSHb*	High
ENSSAUG00010000030	*capn2c*	Low	ENSSAUG00010011634	*CTSK*	High
ENSSAUG00010006640	*capn2d*	High	ENSSAUG00010016582	*CTSLa*	High
ENSSAUG00010000034	*capn2e*	High	ENSSAUG00010010127	*CTSLb*	No
ENSSAUG00010017861	*capn3a*	Low	ENSSAUG00010011634	*CTSLc*	No
ENSSAUG00010012311	*capn3b*	High	ENSSAUG00010002932	*CTSO*	Low
ENSSAUG00010002636	*capn5a*	No	ENSSAUG00010011098	*CTSSa*	High
ENSSAUG00010005676	*capn5b*	High	ENSSAUG00010011632	*CTSSb*	No
ENSSAUG00010025836	*capn6a*	High	ENSSAUG00010011115	*CTSSc*	No
ENSSAUG00010014146	*capn6b*	High	ENSSAUG00010011147	*CTSSd*	No
ENSSAUG00010000033	*capn8a*	Low	ENSSAUG00010017292	*CTSSe*	Low
ENSSAUG00010006205	*capn8b*	No	ENSSAUG00010011128	*CTSSf*	No
ENSSAUG00010026019	*capn8c*	Low	ENSSAUG00010011634	*CTSSg*	No
ENSSAUG00010007897	*capn1*	High	ENSSAUG00010025140	*CTSZa*	High
ENSSAUG00010013017	*capn7*	Low	ENSSAUG00010014606	*CTSZb*	Low
ENSSAUG00010001056	*capn9*	High	ENSSAUG00010010858	*CTSZc*	High
ENSSAUG00010013339	*capn12*	Low	ENSSAUG00010014101	*MMP13a*	High
ENSSAUG00010017388	*capns1a*	High	ENSSAUG00010010684	*MMP13b*	High
ENSSAUG00010002445	*capns1b*	High			

**Table 2:** Genes expressed in the white muscle of European seabass. High: gene expressed in white muscle (logreads > 5), Low: low expression of gene in white muscle (logreads < 5), No: gene not expressed in white muscle.

Gene ID	Gene Name	Expression	Gene ID	Gene Name	Expression
ENSDLAG00005017924	*capn1*	High	ENSDLAG00005013147	*CTSAa*	High
ENSDLAG00005000250	*capn10*	Low	ENSDLAG00005010980	*CTSAb*	Low
ENSDLAG00005001439	*capn11a*	High	ENSDLAG00005004816	*CTSBa*	High
ENSDLAG00005016201	*capn11b*	No	ENSDLAG00005013196	*CTSBb*	No
ENSDLAG00005000961	*capn12*	No	ENSDLAG00005017730	*CTSC*	High
ENSDLAG00005024962	*capn14a*	No	ENSDLAG00005022128	*CTSDa*	High
ENSDLAG00005005672	*capn14b*	Low	ENSDLAG00005004808	*CTSDb*	High
ENSDLAG00005009199	*capn15b*	Low	ENSDLAG00005006074	*CTSF*	High
ENSDLAG00005022265	*capn15a*	No	ENSDLAG00005023385	*CTSH*	High
ENSDLAG00005002296	*capn2b*	High	ENSDLAG00005014479	*CTSK*	High
ENSDLAG00005000590	*capn2a*	No	ENSDLAG00005022121	*CTSLa*	High
ENSDLAG00005015494	*capn3a*	Low	ENSDLAG00005007883	*CTSLb*	No
ENSDLAG00005011625	*capn3b*	High	ENSDLAG00005022875	*CTSO*	Low
ENSDLAG00005005420	*capn5a*	High	ENSDLAG00005005416	*CTSSb*	High
ENSDLAG00005004342	*capn5b*	High	ENSDLAG00005014499	*CTSSa*	Low
ENSDLAG00005001788	*capn6a*	Low	ENSDLAG00005004507	*CTSZa*	High
ENSDLAG00005014943	*capn6b*	High	ENSDLAG00005011006	*CTSZb*	Low
ENSDLAG00005006030	*capn7*	High	ENSDLAG00005026027	*CTSZb.2*	High
ENSDLAG00005000702	*capn8*	High	ENSDLAG00005008130	*MMP13a*	High
ENSDLAG00005018075	*capn9*	High	ENSDLAG00005008348	*MMP13b*	High
ENSDLAG00005012396	*capns1a*	High			
ENSDLAG00005006529	*capns1b*	High			

**Table 3:** Allele frequencies per variant in both species, *Sparus aurata* and *Dicentrarchus labrax*.

Species	SNP ID	Gene	Reference Allele	Alternative Allele	Reference Allele Frequency %	Alternative Allele Frequency %
*S. aurata*	CTSDb_9	*CTSDb*	A	G	61	39
*S. aurata*	capn10_11	*capn10*	T	A	92	8
*S. aurata*	capn10_14	*capn10*	T	A	62	38
*S. aurata*	capn2b_3	*capn2b*	A	T	79	21
*S. aurata*	capn5a_1	*capn5a*	G	A	92	8
*S. aurata*	capn5a_2	*capn5a*	A	G	86	14
*D. labrax*	capn2b_1	*capn2b*	A	C	60	40
*D. labrax*	capn14b_1	*capn14b*	T	A	92	8
*D. labrax*	capn5b_3	*capn5b*	G	T	68	32
*D. labrax*	capn5b_5	*capn5b*	A	G	67	33
*D. labrax*	capn15b_1	*capn15b*	A	G	56	44
*D. labrax*	capn14b_4	*capn14b*	G	A	89	11
*D. labrax*	MMP13b_1	*MMP13b*	G	A	66	34
*D. labrax*	MMP13b_2	*MMP13b*	A	G	79	21
*D. labrax*	MMP13a_1.1	*MMP13a*	T	C	81	19

**Table 4:** Genotypes associated with changes in enzymatic activity in both species, *Sparus aurata* and *Dicentrarchus labrax*. The association was performed using SNPstats. The 95% CI (95% confidence interval), AIC (Akaike information criterion), and BIC (Bayesian information criterion) values were calculated using SPNstats. The model of inheritance with lower AIC and BIC values was selected as the most possible model.

Species	SNP ID	Gene	Alleles	Protein Domain	Aminoacid Change	Model of Inheritance	Genotype	Enzymatic Activity Mean (s.e.)	Enzymatic Activity Difference (95% CI)	*p*-Value	AIC	BIC
*S. aurata*	CTSDb_9	*CTSDb*	A/G	PEPTIDASE_A1	p.Ile314Val	Overdominant	A/A-G/G	17.21 (1.77)	15.70 (7.65–23.75)	0.0002	1247.4	1256.2
							G/A	32.91 (4.76)				
*S. aurata*	capn10_11	*capn10*	T/A	CysPC domain	p.Asp59Val	Dominant	T/T	272.78 (21.31)	−129.97 (−240.56–−19.39)	0.023	2081.3	2090.4
							A/T-A/A	142.8 (36.07)				
*S. aurata*	capn10_14	*capn10*	T/A	CysPC domain	p.Asn3Ile	Recessive	A/A-A/T	275.33 (22.59)	−88.29 (−172.64–−3.93)	0.042	2048.6	2057.6
							T/T	187.04 (34.09)				
*S. aurata*	capn2b_3	*capn2b*	A/T	EF-hand	p.Gln574Leu	Log-additive	---	---	−76.80 (−138.53–−15.07)	0.016	2079.1	2088.1
*S. aurata*	capn5a_1	*capn5a*	G/A	C2 domain	p.Ala414Thr	---	G/G	227.52 (20.28)	109.19 (10.02–208.37)	0.032	2131.9	2141.1
							A/G	336.72 (46.86)				
*S. aurata*	capn5a_2	*capn5a*	A/G	C2 domain	p.Met431Val	Log-additive	---	---	98.30 (1.61–194.99)	0.049	1480.1	1488.1
*D. labrax*	capn2b_1	*capn2b*	A/C	Out of domain	p.Gln12Leu	Dominant	A/A	96.05 (17.34)	−32.23 (−63.79–−0.66)	0.047	1766.7	1775.7
							C/A-C/C	63.82 (7.5)				
*D. labrax*	capn14b_1	*capn14b*	T/A	CysPC domain	p.Ser118Pro	Recessive	T/T-A/T	71.4 (7.07)	153.77 (53.06–254.48)	0.0032	1808.7	1817.7
							A/A	225.17 (96.73)				
*D. labrax*	capn5b_3	*capn5b*	G/T	CysPC domain	p.Gly227Cys	Dominant	G/G	44.03 (7.96)	52.20 (23.69–80.71)	0.0005	1520.8	1529.4
							G/T-T/T	96.23 (11.61)				
*D. labrax*	capn5b_5	*capn5b*	A/G	C2 domain	p.Met388Val	Overdominant	A/A-G/G	94.07 (10.91)	−48.39 (−78.47–−18.31)	0.002	1643.8	1652.6
							G/A	45.68 (8.68)				
*D. labrax*	capn15b_1	*capn15b*	A/G	Zinc finger	p.Ser21Gly	Dominant	A/A	96.34 (17.35)	−38.72 (−75.58–−1.86)	0.042	1047.6	1055.1
							A/G-G/G	57.62 (10.2)				
*D. labrax*	capn14b_4	*capn14b*	G/A	Out of domain	p.Ala357Thr	Recessive	G/G-A/G	71.29 (7.17)	107.14 (18.49–195.79)	0.019	1788.9	1798
							A/A	178.43 (82.84)				
*D. labrax*	MMP13b_1	*MMP13b*	G/A	Catalytic domain	p.Gly103Arg	Overdominant	G/G-A/A	27.69 (3.01)	−13.59 (−20.16–−7.02)	0.0001	1304.1	1313.1
							A/G	14.1 (1.81)				
*D. labrax*	MMP13b_2	*MMP13b*	A/G	Peptidoglycan binding-like	p.Asn34Ser	Overdominant	A/A-G/G	26.82 (2.87)	−15.55 (−24.92–−6.17)	0.0016	895.7	903.5
							G/A	11.28 (2.99)				
*D. labrax*	MMP13a_1.1	*MMP13a*	T/C	Peptidoglycan binding-like	p.Ser26Gly	Recessive	T/T-T/C	18.13 (1.71)	24.41 (7.98–40.84)	0.0042	1259.1	1268
							C/C	42.54 (9.61)				

**Table 5:** Sift algorithm results. The sift score is indicative of the amino acid substitution effect on the protein. A threshold of <0.05 exists for non-tolerated mutations. Results with underlined bold font depict the mutations that are predicted to affect protein function. Results with an asterisk (*) depict the mutations that are predicted to affect protein function but with low confidence.

Species	SNP ID	Mutation	SIFT Score
*S. aurata*	capn5a_1	p.Ala414Thr	**0.01**
*S. aurata*	capn5a_2	p.Met431Val	0.86
*S. aurata*	capn2b_3	p.Gln574Leu	1
*S. aurata*	capn10	p.Met254Lys	0.63
*S. aurata*	capn10_11	p.Asp59Val	**0.03**
*S. aurata*	CTSDb_9	p.Ile314Val	0.27
*D. labrax*	capn2b_1	p.Gln12Leu	0.01 *
*D. labrax*	capn5b_3	p.Gly227Cys	0.68
*D. labrax*	capn5b_5	p.Met388Val	0.66
*D. labrax*	capn14b_4	p.Ala357Thr	0.71
*D. labrax*	capn14b_1	p.Ser118Pro	0.26
*D. labrax*	capn15b_1	p.Ser21Gly	0.02 *
*D. labrax*	MMP13a	p.Ser26Gly	0.42
*D. labrax*	MMP13b_1	p.Gly103Arg	**0.02**
*D. labrax*	MMP13b_2	p.Asn34Ser	0.48

## Data Availability

All data are provided in this article.

## Supplementary Materials

The following supporting information can be downloaded at https://www.mdpi.com/article/10.3390/ani14020205/s1; Supplementary Material S1: List of primer tables for each species and tables with genotype frequencies and response averages for each genotype; Supplementary Material S2: List of figures with the enzymatic activities for each genotype.
