# Supplementary material for: MassArray Genotyping as a Selection Tool for Extending the Shelf-Life of Fresh Gilthead Sea Bream and European Seabass
Source: Animals (Basel). 2024 Jan 8;14(2):205. doi: 10.3390/ani14020205 (PMC10812826; doi:10.3390/ani14020205)
Supplement: Supplementary file 1 [file animals-14-00205-s001.zip › Supplementary Material S1.pdf]

Supplementary Table S1. Primers used for genotyping each SNP along with chromosome and SNP position in Gilthead sea bream

Gene	SNP	Allele	Chromosome	SNP Position	Forward primer	Reverse primer	Extension primer
CAPN12	SA_CAPN12_1	T/C	2	14,392,434	ACGTTGGATGACAGCAGGACAAGGCAGAG	ACGTTGGATGCCTTCTGTTTCTCAGCTATC	GGAGTAGGAGGAGATGACCC
CAPN1	SA_CAPN1_2	C/G	10	17,013,632	ACGTTGGATGGTACAGGCCTTTGTTTTGGG	ACGTTGGATGCTTAGCTATGCTAGCTAATG	TAATGTAGCTTGTTTCTAGTCTT
CAPN1	SA_CAPN1_3	G/C	10	17,014,847	ACGTTGGATGTATGTTACAGGTAGCGTCAG	ACGTTGGATGGATCTTGCTCAATACTGGAC	GTAACATTAGATACTTGGACACAAAA
CAPN10	SA_CAPN10_10	C/T	21	25,509,752	ACGTTGGATGCCTGCTGCCAGAAACTAAAC	ACGTTGGATGTGACTCTCTGTAGGTGTTGC	AAGACTCAGTGGGGTGACAGCA
CAPN10	SA_CAPN10_11	T/A	21	25,509,996	ACGTTGGATGGTTTCACATGAGCCAGGTTG	ACGTTGGATGGTCGTCTGATGATTGTGTCC	ATGAGCCAGGTTGATGTTG
CAPN10	SA_CAPN10_14	T/A	21	25,510,883	ACGTTGGATGGGTGTGTATGTGCATGTTTC	ACGTTGGATGGTGACACTATATGTGTGTATG	CCCCCAAACAATGTAAGTTCACCA
CAPN10	SA_CAPN10_2	G/A	21	25,505,560	ACGTTGGATGACATCCTGGGTGTAGCAGTG	ACGTTGGATGTGCATCCAGTCTCTAGAAGG	GGGACGATGATCCTGTGG
CAPN10	SA_CAPN10_3	A/G	21	25,506,230	ACGTTGGATGTAGAGCCGTGTGTGTACGTC	ACGTTGGATGCCTTCTCTCGAGGTCACAAG	CATTTGTACGTCACGGCCC
CAPN10	SA_CAPN10_4	A/T	21	25,506,914	ACGTTGGATGACACTGTAAAAGGGAAGCGG	ACGTTGGATGAGAACACTTCCTTCAGTGGC	TCAGTGGCTCGTGGG
CAPN10	SA_CAPN10_5	G/A	21	25,508,216	ACGTTGGATGACTTAGGGTTGCTGCTGTAG	ACGTTGGATGTGTTGACACACAGCCATCAG	TTTACGGCTGCCGCCC
CAPN10	SA_CAPN10_7	A/T	21	25,509,126	ACGTTGGATGGCCGTACTTCACTTCCTGAC	ACGTTGGATGTTGGTGACTGTTCTAGGTGC	CCCGGGAGTGGCTGGATGTGA
CAPN10	SA_CAPN10_8	C/A	21	25,509,391	ACGTTGGATGTCTTTCACTGGGTGCAGAAG	ACGTTGGATGTGGAGCTTGGGAGACTTTGG	GGGGATGTTCTGGTCTCTGCTC
CAPN10	SA_CAPN10_9	G/A	21	25,509,636	ACGTTGGATGCTTACTTGGCATAGGCCTTC	ACGTTGGATGGTATCAACTCCACCCTTTGC	TCGCGCTGCCACTCC
CAPN11b	SA_CAPN11b_2	A/G	1	607,073	ACGTTGGATGTTTGAGGACCCAATGTTCCC	ACGTTGGATGTTACCATCGGTCTCTTCCAC	GACCATGTTCCCTGCAGAACCT
CAPN14	SA_CAPN14_1	A/T	16	19,939,536	ACGTTGGATGGACATTCCAGTCTGACATCC	ACGTTGGATGTTACCCTGAAGTGGAACAGC	TCCCAGTTGTTCCTCTTGACC
CAPN15	SA_CAPN15_6	C/A	23	28,984,847	ACGTTGGATGTTCACCAGCTGCACTGTTTC	ACGTTGGATGTGACCTTGTTGATGAGGGTG	CACACTCCTCCGTCCTGT
CAPN15	SA_CAPN15_8	A/C	23	28,985,779	ACGTTGGATGTCCAACATCGGGTCAGAAAG	ACGTTGGATGCGGAAGACAGACCACTTCAC	GGTTGCAGCAGTTGAT
CAPN2b	SA_CAPN2b_2	G/T	15	122 28 009	ACGTTGGATGACAAAGAGGTTCTGTGTCGC	ACGTTGGATGCGAAGTTCTGTACCTCGAAG	GACGTGTCGAAGCTTTGGT
CAPN2b	SA_CAPN2b_3	A/T	15	122 31 672	ACGTTGGATGTGGTTCATTTTCAGGATCGG	ACGTTGGATGAGGAAGGATACATCCAGCAG	ATCTGATGTAAAAAACAATGGCTTT
CAPN2b	SA_CAPN2b_4	A/T	15	122 31 676	ACGTTGGATGTGGTTCATTTTCAGGATCGG	ACGTTGGATGAGGAAGGATACATCCAGCAG	AGGGTGATCATGTTGCGGCAAGTCGTT
CAPN5a	SA_CAPN5a_1	G/A	2	2,929,051	ACGTTGGATGGTTTCCATAAGACCTGGGAG	ACGTTGGATGGGAGTGCTTGTTGTTGATGC	CCGCTTCTCAACCGA
CAPN5a	SA_CAPN5a_2	A/G	2	2,929,209	ACGTTGGATGCTCTACCAGTACTGAGTGTG	ACGTTGGATGAGACAGATCAGCACCTCATC	CCTCTCTGGTGTAGTTC
CAPN5b	SA_CAPN5b_1	A/T	13	26,143,793	ACGTTGGATGATTCGTTGTAGAGAGAGGGC	ACGTTGGATGGGCAAAAGTACCTCAGTGTC	GTGAGGGCAGAAATTTGAAAAAACA
CAPN6	SA_CAPN6_2	C/T	10	20,905,979	ACGTTGGATGTACACTTCTACCCAAAATGC	ACGTTGGATGATAAGGATTGGGGCCATCTG	GAAATTCTACCCAAAATGCTTATATA

CAPN6	SA_CAPN6_3	T/C	10	20,907,325	ACGTTGGATGCCTAATGTGAATCCCTGCTG	ACGTTGGATGCTGAGTGGAGCATTAGGAAC	GCTTGAGAAAAAGCGGGT
CAPN6	SA_CAPN6_4	C/T	10	20,907,500	ACGTTGGATGCACTCTGTACTGTCTTGTTC	ACGTTGGATGAGGCAAGATGTGAACCTGTC	GTCCTAGGCTGCCTAGATTGGGAAGT
CAPN6	SA_CAPN6_5	T/G	10	20,907,603	ACGTTGGATGGTCAAAAGTCAATAATCAGGG	ACGTTGGATGCCTGATTTTGGGCTATGACG	CAATAATGTAAAAGTATGGTTGA
CAPN6	SA_CAPN6_6	C/T	10	20,907,688	ACGTTGGATGGCGTACAGAAATGGGTACAG	ACGTTGGATGCAGGCAACGTACAACAGGTC	GAACAAGTACACAATTATTTGAA
CAPN7	SA_CAPN7_1	C/T	17	35,589,374	ACGTTGGATGGTCCTTCCTGTTTTGGCAAG	ACGTTGGATGATGTGGCTTCTGCTACATGG	AGGGGACATGGAGGCGGAACAC
CAPN7	SA_CAPN7_2	C/G	17	35,591,003	ACGTTGGATGACCTGTAATCTCCACTGCTC	ACGTTGGATGACAGCGTTGGGTTTGAGATG	TAATATCCACTGCTCTTTTTCTGGAAGG
CAPN7	SA_CAPN7_3	A/T	17	35,595,648	ACGTTGGATGTTTTTGTGGCTGCTCTCTAC	ACGTTGGATGAACTACACACTGAGGGTGAG	GATCCAATTTGTTGTAAAAAAAACAAAC
CAPN7	SA_CAPN7_8	C/G	17	35,601,824	ACGTTGGATGCCAAGAGGGAAAAACTGCCG	ACGTTGGATGATCAACTCCAGCTCAGACTC	GGAGACGGCCGCACTGCTGG
CAPN9	SA_CAPN9_6	C/T	1	4,170,399	ACGTTGGATGAGCCTAGTTTGCTTTTTCAC	ACGTTGGATGTTCTCTGAGAAGAAGGCCAC	GCTCTGTGAGTGCTC
CAPN9	SA_CAPN9_7	G/C	1	4,176,881	ACGTTGGATGACCTTTGGCCTCTTCCATTC	ACGTTGGATGTTTGAGGATCCAGACTTCCC	CCTCGCTCTTCTTCAG
CTSA	SA_CTSA_10	G/T	6	131,924	ACGTTGGATGTGCTGTCTCTGTTCCATTTC	ACGTTGGATGCAGAAGAAAATACATGTCAGG	GGACATACATGTCAGGATGACAA
CTSA	SA_CTSA_11	T/C	6	133,137	ACGTTGGATGTCTCTAGATGACGTGATGGG	ACGTTGGATGGTTGGTACATTACCTCTGCC	GCAGCACGTCTCAGG
CTSA	SA_CTSA_8	C/T	6	125,458	ACGTTGGATGAGTTCAGACATTGGCCAGAG	ACGTTGGATGATTGTGTGGTTGATGCGGAG	ACAGGGTTGATGCGGAGCAGGAAGT
CTSA	SA_CTSA_9	G/A	6	126,061	ACGTTGGATGAAAATCCCAGGTCTGCTGTC	ACGTTGGATGGATTGCTCTTAGCGATGGTG	CCGTCACGCCCCAGCAA
CTSB	SA_CTSB_2	G/A	4	30,128,431	ACGTTGGATGATGACTGTGACACAGCAGAG	ACGTTGGATGGGCTGGAAATTCGGTTTTAG	AACAGCAGAGGACTGGA
CTSB	SA_CTSB_3	G/A	4	30,128,431	ACGTTGGATGATGACTGTGACACAGCAGAG	ACGTTGGATGGGCTGGAAATTCGGTTTTAG	CTGCCACAGCAGAGGACTGGA
CTSDb	SA_CTSDb_8	A/G	4	37,872,369	ACGTTGGATGCTGACGCAGAACACAACTAC	ACGTTGGATGCCAACTACAATCGACATGAC	ATCGACATGACGATAAAAGCA
CTSZa	SA_CTSZa_2	A/C	18	36,645,823	ACGTTGGATGTTTCTGTCCAACACGTGGTC	ACGTTGGATGCATGTTTGTGAGCGTACTCC	GGGCCCCCTGCCGTGGTCTCCCCC
CTSZa	SA_CTSZa_3	C/G	18	36,646,117	ACGTTGGATGTTTGGAGCCATTAGTGGACG	ACGTTGGATGGTACAGCTCTCCAAAAGGTC	TGATGGCAGAAATCTACA
CTSZb	SA_CTSZb_1	T/G	21	15,413,453	ACGTTGGATGCTGTGCATATGGAGACACTA	ACGTTGGATGGAACTGTTCCTTTACTTTGC	CCCCTGGAGACACTATTGTTACT
mmp13a	SA_MMP13a_7	C/T	13	17,216,954	ACGTTGGATGGACACCAGCATTAAAATGTCC	ACGTTGGATGAAGGTGCTGTTCTCAGGAAG	CCCCGTCTCAGGAAGGCCATATAACCA
mmp13a	SA_MMP13a_8	A/C	13	17,217,153	ACGTTGGATGATTGCACATGTGAAGATGTC	ACGTTGGATGGCTACATAATAACAAAACAC	GAAATGTGATTGAACAATGTTTAT
mmp1a	SA_MMP1a_1	A/G	2	24,797,740	ACGTTGGATGATCTTCCATTCAACCACGAC	ACGTTGGATGTTAATGCTTGGGCCTTCAAC	TCTTATCCCTGACCGATTT
mmp2	SA_MMP2_1	C/T	4	34,545,888	ACGTTGGATGGTCACATCCGTCTTCGTTTG	ACGTTGGATGTGCAATAACATCTCTGTGCC	TGGATATGAGAAACAGAAGAGTATG
mmp2	SA_MMP2_3	C/A	4	34,552,742	ACGTTGGATGTTAAAAGTAGGATCCCCCCC	ACGTTGGATGGCTTCGAGAATGACTCACTG	TTGTCCGACATCACAAATAT
mmp13a	SA_MMP13a_10	C/A	13	17,217,971	ACGTTGGATGCCCGAAATGCCAAATCTGAC	ACGTTGGATGGTACAAGATCATAGCCACTG	AATGCCTGCAATATTACCTTAACATGC
mmp13a	SA_MMP13a_11	T/A	13	17,218,019	ACGTTGGATGCCCGAAATGCCAAATCTGAC	ACGTTGGATGGTACAAGATCATAGCCACTG	CCAGCATAGCCACTGAAAGCCC

mmp13a	SA_MMP13a_17	C/T	13	17,224,350	ACGTTGGATGGGCATCTCAGCTTTACCTTC	ACGTTGGATGAAAGGCAAATCAGAGTTGGG	TGCCTTGAATCTCGTTG
mmp13a	SA_MMP13a_18	G/C	13	17,224,530	ACGTTGGATGGGCATCTCAGCTTTACCTTC	ACGTTGGATGAAAGGCAAATCAGAGTTGGG	CGTTGGGTTTACAAAATTAATCTAT
mmp13a	SA_MMP13a_20	A/C	13	17,225,551	ACGTTGGATGATGCCCCCATCAACATTGAC	ACGTTGGATGCGCTTTGGGTAAATGCAGAG	CCAAACAAGAACTAGACAATGTCTT
mmp13a	SA_MMP13a_21	G/T	13	17,225,553	ACGTTGGATGACATCTACAGTGGACCCTAC	ACGTTGGATGTAGAAGTTAGTGCAGCGCAG	AGCCGCTTGCTCCTCAGGCTGTACT
mmp13a	SA_MMP13a_6	T/C	13	17,216,956	ACGTTGGATGACCCAACTCTGATTTGCTTC	ACGTTGGATGCACCGTGTGCTGTTAGTAAG	GATTTGCTTCTTTTAACACAA
mmp13a	SA_MMP13a_9	G/T	13	17,217,178	ACGTTGGATGGGATGAGACCTTCACTTTCC	ACGTTGGATGACACTGACAGATTCATTACC	TATTTATGCTCTGCCTAGT

Supplementary Table S2. Primers used for genotyping each SNP along with chromosome and SNP position in European seabass

Gene	SNP	Allele	Chromosome	SNP Position	Forward primer	Reverse primer	Extension primer
CAPN10	DL_Capn10_1	G/T	LG10	10,757,774	ACGTTGGATGTCGACCCTGCTTCTGCTTTG	ACGTTGGATGCAGTCTCATCCAACCAGAAC	AGACCTCGCCCTCGGCCACC
CAPN10	DL_Capn10_2	G/A	LG10	10,759,261	ACGTTGGATGAACAACTGATGGAGAGTGGG	ACGTTGGATGAGGAAGTTCCTGCTTCCTCC	GGAGACCAGTTACTTCC
CAPN10	DL_Capn10_3	T/C	LG10	10,760,016	ACGTTGGATGACTTATCAGCCTGACTGCTC	ACGTTGGATGGAAAAAGAAACGGAGCCTAC	GGGCCGGAGCCTACCTGTGT
CAPN2A	DL_CAPN2A_1	T/A	LG11	6,391,234	ACGTTGGATGTACAAGCACCTTGCTAACCC	ACGTTGGATGGACAAACTGAGGGTTCATCC	CTACCCCTTGCTTCACTTCCTAGAT
CAPN2a	DL_CAPN2A_2	C/G	LG11	6,393,433	ACGTTGGATGGGACTTCACTGGAGGCATTG	ACGTTGGATGTCTCCAAGGCCTTCTTTATG	AGGCATTGCTGAGAG
CAPN2B	DL_CAPN2B_3	C/T	LG11	6,396,625	ACGTTGGATGGGCTTCAACATCAACATCGC	ACGTTGGATGATAGCATACTGAGCGTCAGC	CCACTCGCTGTGCTGCAGG
CAPN2b	DL_CAPN2B_4	G/T	LG11	6,397,796	ACGTTGGATGAGGACATGGAGGTATCAGTC	ACGTTGGATGTCGGAGATTGATGTACTCAC	CAACTTTTGAACAACGTTGTC
CAPN2B	DL_CAPN2B_1	A/C	LG11	6,399,462	ACGTTGGATGGATCCAATGAATGGAACTACG	ACGTTGGATGCCAGAACTCTCCATCTTCAG	GTTCAACTTTGATTTCTCATC
CAPN2B	DL_CAPN2B_2	A/T	LG11	6,401,916	ACGTTGGATGATGACGTCCACGGCAGACC	ACGTTGGATGTTGGTGCCAATGCCTTGTTC	CCTTGTTCCCTCTTC
CAPN14B	DL_CAPN14B_1	T/A	LG12	6,663,157	ACGTTGGATGTGACTGTTCTGCAGAACAGG	ACGTTGGATGTCCTCTTGTCAGGTTTTTGC	TCTTCAGAACAGGGGGAAAA
capn14b	DL_capn14b_4	G/A	LG12	6,663,879	ACGTTGGATGATGGAAGCACTTCTTGCCAC	ACGTTGGATGATTCATGCATCCTCCAGCAG	CTTGCCACTGGAAG
capn14b	DL_capn14b_5	T/C	LG12	6,681,556	ACGTTGGATGTGGTCAGATGATGAGCCAAG	ACGTTGGATGATTCCACTCTCCTTTTCCCC	GTGAGCCAAGGGAAAG
CAPN14B	DL_CAPN14B_2	C/A	LG12	6,681,566	ACGTTGGATGGTCTCCCATGATGATTGCTG	ACGTTGGATGCACAAACGCACCAGGTTTAC	CCTAGGTTTACCACTTTCCCTT
CAPN14B	DL_CAPN14B_3	T/C	LG12	6,684,965	ACGTTGGATGCTGGTTTCTTGCGTCTATCG	ACGTTGGATGTTCAAGAGGGACAACTTGCC	GGTGCTTTGACGTTCCAG
capn12	DL_CAPN12_1	A/G	LG13	18,103,034	ACGTTGGATGGGAAGAATCCTCAGTTCCAG	ACGTTGGATGTCATCGTCGTTACTTTCCTC	TTTTCTCACAGAGCAGGAC
capn5b	DL_CAPN5b_7	A/G	LG13	27,344,383	ACGTTGGATGCCCTACGTGATCATTCGTTG	ACGTTGGATGGGGCTGCGTGTGTTTTTATG	GTTTTTTTATGGACCGGAGAG
capn5b	DL_CAPN5b_8	G/A	LG13	27,345,279	ACGTTGGATGTTGGTGGGAGCGTCTACATC	ACGTTGGATGCCCCTCTTTCAGGTCGATTC	ATTCGTAAGAACACAGAC
capn5b	DL_CAPN5b_9	G;/A	LG13	27,345,919	ACGTTGGATGCATGTTTGATGTGAAGAAGCC	ACGTTGGATGTTGGCTCTGCGGTCTTTCTG	ATGGGAAGAAGCCGGAGGA
CAPN5b	DL_CAPN5b_7	A/T	LG13	27,345,945	ACGTTGGATGGAAGAAGGAAACAACTGTAAC	ACGTTGGATGGTCCTCCGGCTTCTTCACAT	GCTTCTTCACATCAAACA
CAPN5b	DL_CAPN5b_1	G/A	LG13	27,346,973	ACGTTGGATGCCAACCTGAGTTTCCGTAAG	ACGTTGGATGTTTTGCTGGTCGTCGTGTTG	CAGCATGGCTTCCT
CAPN5b	DL_CAPN5b_2	G/T	LG13	27,347,818	ACGTTGGATGACAGAGTCCTGAAAGTCCAC	ACGTTGGATGTGTCTATTCTCTCCTCACCC	GTTATCTCCTCACCCGGATTGAGC
capn5b	DL_CAPN5b_10	G/C	LG13	27,347,866	ACGTTGGATGTGGACTTGAGGGAGAAGATG	ACGTTGGATGGTTGTGGACTTTCAGGACTC	CCCCAAGGACTCTGTCAAACA
CAPN5b	DL_CAPN5b_3	G/T	LG13	27,347,920	ACGTTGGATGTCTCATGGATTTCACCGGAG	ACGTTGGATGTCGCGTTGCTCTTCATCTTC	CTCCCTCAAGTCCATTG
CAPN5b	DL_CAPN5b_4	C/G	LG13	27,347,970	ACGTTGGATGTAGGATGTACGGCTGCTATG	ACGTTGGATGTCCGGTGAAATCCATGAGAG	TCTCGTCGGCCGTGTTGCCC

CAPN5b	DL_CAPN5b_5	A/G	LG13	27,348,369	ACGTTGGATGTTCTGGCGGTTTGGTGAGTG	ACGTTGGATGTACACCAGCTCTCCGTTCAC	CCCCGGTCATCGATCACCA
CAPN5b	DL_CAPN5b_6	G/A	LG13	27,349,364	ACGTTGGATGTTTGAAGACCCTCTGTTCCC	ACGTTGGATGTTTTCCAGGTGACTCTGCCG	CCCCCACCAGTCTCTGTTCTACC
MMP13a	DL_MMP13a_1	C/T	LG13	9,146,897	ACGTTGGATGTAATGGTCTCTCCTCTGAGC	ACGTTGGATGCCTGACGCACCTGACAAATG	GGGGTGACAAATGTGACCCCAT
MMP13a	DL_MMP13a_1.1	T/C	LG13	9,148,571	ACGTTGGATGCTTGTCCCGAAATGCATCAG	ACGTTGGATGACGTCGGTTCTATGACCTTC	TTCCAGCTGGTCTCCAA
MMP13b	DL_MMP13B_4	G/A	LG14	6,894,856	ACGTTGGATGAACGTTGCCAGTTTCTCCAC	ACGTTGGATGTTCACCTGTAGGTGAGTCTG	TTTCTCCCATTTGAGG
MMP13b	DL_MMP13b_1	G/A	LG14	6,904,296	ACGTTGGATGCTACTAGGTTTTAACTTCTCC	ACGTTGGATGCGGTCTCCTCTGTTAGGTTG	ATGTTTTAACTTCTCCTTTCAGA
MMP13b	DL_ MMP13b_2	A/G	LG14	6,904,502	ACGTTGGATGACCAACGTTGCCAGTTTCTC	ACGTTGGATGTTCACCTGTAGGTGAGTCTG	GGGGATCCCATTTGGGGCTATTTC
MMP13b	DL_ MMP13b_3	G/C	LG14	6,904,774	ACGTTGGATGAAGAATCTACAGTGGCACCG	ACGTTGGATGCCACCACTGAGAGTTATTTG	GATAGTTATTTGGTACTCACATCGG
CAPN15B	DL_CAPN15B_1	A/G	LG1B	5,989,368	ACGTTGGATGGTGGACGCTGCACCTTCTTA	ACGTTGGATGAAGGTCAGGTTTTTGGCGTG	GGAAGCTGCACCTTCTTAAATGCC
CAPN15B	DL_CAPN15B_2	A/G	LG1B	5,990,283	ACGTTGGATGAGAAGACTCTCAGCACCATC	ACGTTGGATGATGAGCAGCTAGGGCACTTC	GGAGGTTGCTTCCTCAGTC
CTSLa	DL_CTSLa_1	G/A	LG4	20,590,166	ACGTTGGATGCTTATGTGATGTTCTTGTCCC	ACGTTGGATGCACAGCACAACAAAGAGCAG	GAGGCTGATCTGCTG
CTSLa	DL_CTSLa_3	A/G	LG4	20,590,173	ACGTTGGATGATGTTCTTGTCCCTGTAGTG	ACGTTGGATGCACAGCACAACAAAGAGCAG	AGCAGGAGGCTGA
CTSLa	DL_CTSLa_2	G/T	LG4	20,591,349	ACGTTGGATGAATTTGCGAAGAGAGGGAGG	ACGTTGGATGCCACAACCCAAGCATCTAAC	TAGGGAGGATTGTCTCG
CTSBa	DL_CTSBa_1	G/A	LG5	10,442,028	ACGTTGGATGAGTATGCTGGAGGCATGAAG	ACGTTGGATGTCTGATCTCCTTCAGAGTGG	TCTCGGGAGTCGAAC
CTSBa	DL_CTSBa_2	G/A	LG5	10,444,013	ACGTTGGATGGCCATGCCATCAAGATCCTG	ACGTTGGATGAGTCAGTGTTCCAGGAGTTG	GCCAGTAGGGAACACCA
CTSBa	DL_CTSBa_3	A/T	LG5	27,041,753	ACGTTGGATGACCCTGCGGTCAGCTGATAA	ACGTTGGATGCGACAATGACGGAGCACTTC	GGGCTGTTGTCGGGAAAC
CTSHb	DL_CTSHb_1	G/A	LG6	10,932,853	ACGTTGGATGCCTCCCCAGTCAAGCATTTG	ACGTTGGATGGCTGTGTATGGGTAGTCTTC	AAGTCAAGCATTTGAATACATC
CTSHb	DL_CTSHb_2	T/G	LG6	10,934,050	ACGTTGGATGAGACAAGGTGAACCATGCTG	ACGTTGGATGCACTATCCAGTAAGGAGAGC	AAGTAAGGAGAGCCATTTTC
CAPN7	DL_CAPN7_1	T/C	UN	71,744,391	ACGTTGGATGCTTCTGCACTAATCTTGTCC	ACGTTGGATGACTCATGAAGGGCACATAGG	TCAGGAGTACATCTACAATCAAT
CAPN7	DL_CAPN7_2	G/A	UN	71,756,202	ACGTTGGATGTTCTTGGACTTTGCCAGCAC	ACGTTGGATGCTCTCTCCAGCTCCATCTTC	CCTCAAGGTCTCCCAG

Supplementary Table S3. SA_CTSDb_8_9 genotype frequencies and response averages for each genotype

SA_CTSDb_8_9 genotype frequencies (n=161)		
Genotype	Proportion	Response mean (s.e.)
A/A	0.46	15.55 (1.88)
A/G	0.29	32.91 (4.76)
G/G	0.25	20.33 (3.68)

Supplementary Table S4. SA_CAPN10_ genotype frequencies and response averages for each genotype

SA_CAPN10_11 genotype frequencies (n=161)		
Genotype	Proportion	Response mean (s.e.)
A/A	0.02	136.6 (93.25)
T/A	0.11	143.9 (40.32)
T/T	0.87	272.78 (21.31)

Supplementary Table S5. SA_CAPN10_14 genotype frequencies and response averages for each genotype

SA_CAPN10_14 genotype frequencies (n=161)		
Genotype	Proportion	Response mean (s.e.)
A/A	0.5	282.14 (28.23)
A/T	0.24	261.33 (37.82)
T/T	0.26	187.04 (34.09)

***Supplementary* Table S6**. SA_CAPN2b_3 genotype frequencies and response averages for each genotype

SA_CAPN2b_3 genotype frequencies (n=161)		
Genotype	Proportion	Response mean (s.e.)
A/A	0.06	117.33 (47)
T/A	0.3	207.33 (29.68)
T/T	0.64	279.53 (25.92)

Supplementary Table S7. SA_CAPN5a_1 genotype frequencies and response averages for each genotype

SA_CAPN5a_1 genotype frequencies (n=161)		
Genotype	Proportion	Response mean (s.e.)
G/A	0.16	336.72 (46.86)
G/G	0.84	227.52 (20.28)

Supplementary Table S8. SA_CAPN5a_2 genotype frequencies and response averages for each genotype

SA_CAPN5a_2 genotype frequencies (n=161)		
Genotype	Proportion	Response mean (s.e.)
A/A	0.01	512.1 (0)
G/A	0.26	326.52 (51.3)
G/G	0.73	235.07 (25.9)

Supplementary Table S9. DL_CAPN2B_1 genotype frequencies and response averages for each genotype

DL_CAPN2B_1 genotype frequencies (n=185)		
Genotype	Proportion	Response mean (s.e.)
A/A	0.32	96.05 (15.65)
A/C	0.57	63.57 (7.91)
C/C	0.11	64.99 (16.41)

Supplementary Table S10. DL_CAPN14B_1 genotype frequencies and response averages for each genotype

DL_CAPN14B_1 genotype frequencies (n=185)		
Genotype	Proportion	Response mean (s.e.)
A/A	0.02	225.17 (83.77)
T/A	0.12	45.94 (12.88)
T/T	0.86	74.87 (7.29)

Supplementary Table S11. DL_CAPN5b_3 genotype frequencies and response averages for each genotype

DL_CAPN5b_3 genotype frequencies (n=185)		
Genotype	Proportion	Response mean (s.e.)
G/G	0.51	44.03 (7.12)
G/T	0.34	98.61 (14.18)
T/T	0.16	91.36 (19.24)

Supplementary Table S12. DL_CAPN5b_5 genotype frequencies and response averages for each genotype

DL_CAPN5b_5 genotype frequencies (n=185)		
Genotype	Proportion	Response mean (s.e.)
A/A	0.48	94.18 (11.7)
A/G	0.38	45.68 (8.3)
G/G	0.14	93.41 (20.44)

Supplementary Table S13. DL_CAPN15B_1 genotype frequencies and response averages for each genotype

DL_CAPN15B_1 genotype frequencies (n=185)		
Genotype	Proportion	Response mean (s.e.)
A/A	0.33	96.34 (16.83)
A/G	0.46	45.97 (9.77)
G/G	0.21	80.91 (19.51)

Supplementary Table S14. DL_capn14b_4 genotype frequencies and response averages for each genotype

DL_capn14b_4 genotype frequencies (n=185)		
Genotype	Proportion	Response mean (s.e.)
A/A	0.03	178.43 (74.09)
G/A	0.16	40.05 (10.09)
G/G	0.81	77.09 (7.73)

Supplementary Table S15. DL_MMP13b_1 genotype frequencies and response averages for each genotype

DL_MMP13b_1 genotype frequencies (n=185)		
Genotype	Proportion	Response mean (s.e.)
A/A	0.05	26.67 (12.55)
G/A	0.58	14.1 (1.67)
G/G	0.37	27.83 (2.85)

Supplementary Table S16. DL_MMP13b_2 genotype frequencies and response averages for each genotype

DL_MMP13b_2 genotype frequencies (n=185)		
Genotype	Proportion	Response mean (s.e.)
A/A	0.64	26.33 (2.59)
A/G	0.31	11.28 (2.77)
G/G	0.05	32.03 (16.46)

Supplementary Table S17. DL_MMP13a_1.1 genotype frequencies and response averages for each genotype

DL_MMP13a_1.1 genotype frequencies (n=185)		
Genotype	Proportion	Response mean (s.e.)
C/C	0.07	42.54 (7.1)
T/C	0.24	20.27 (4.31)
T/T	0.69	17.58 (1.71)
