# Supplementary material for: MassArray Genotyping as a Selection Tool for Extending the Shelf-Life of Fresh Gilthead Sea Bream and European Seabass
Source: Animals (Basel). 2024 Jan 8;14(2):205. doi: 10.3390/ani14020205 (PMC10812826; doi:10.3390/ani14020205)
Supplement: Supplementary file 1 [file animals-14-00205-s001.zip › Supplementary Material S2.pdf]

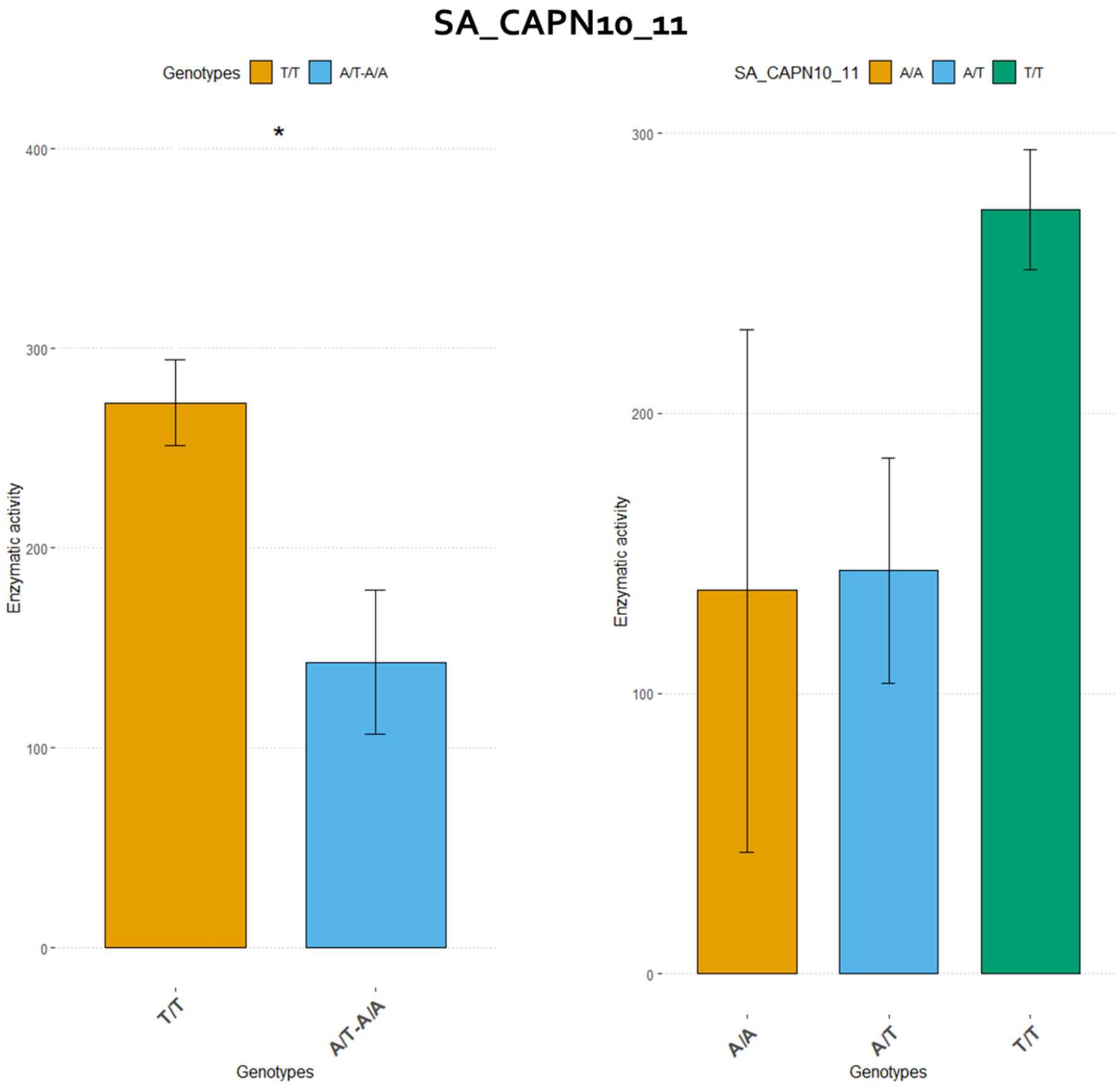


**Figure S1.** Enzymatic activity of Calpains by each genotype. SA_CAPN10_11 has a dominant model of inheritance.

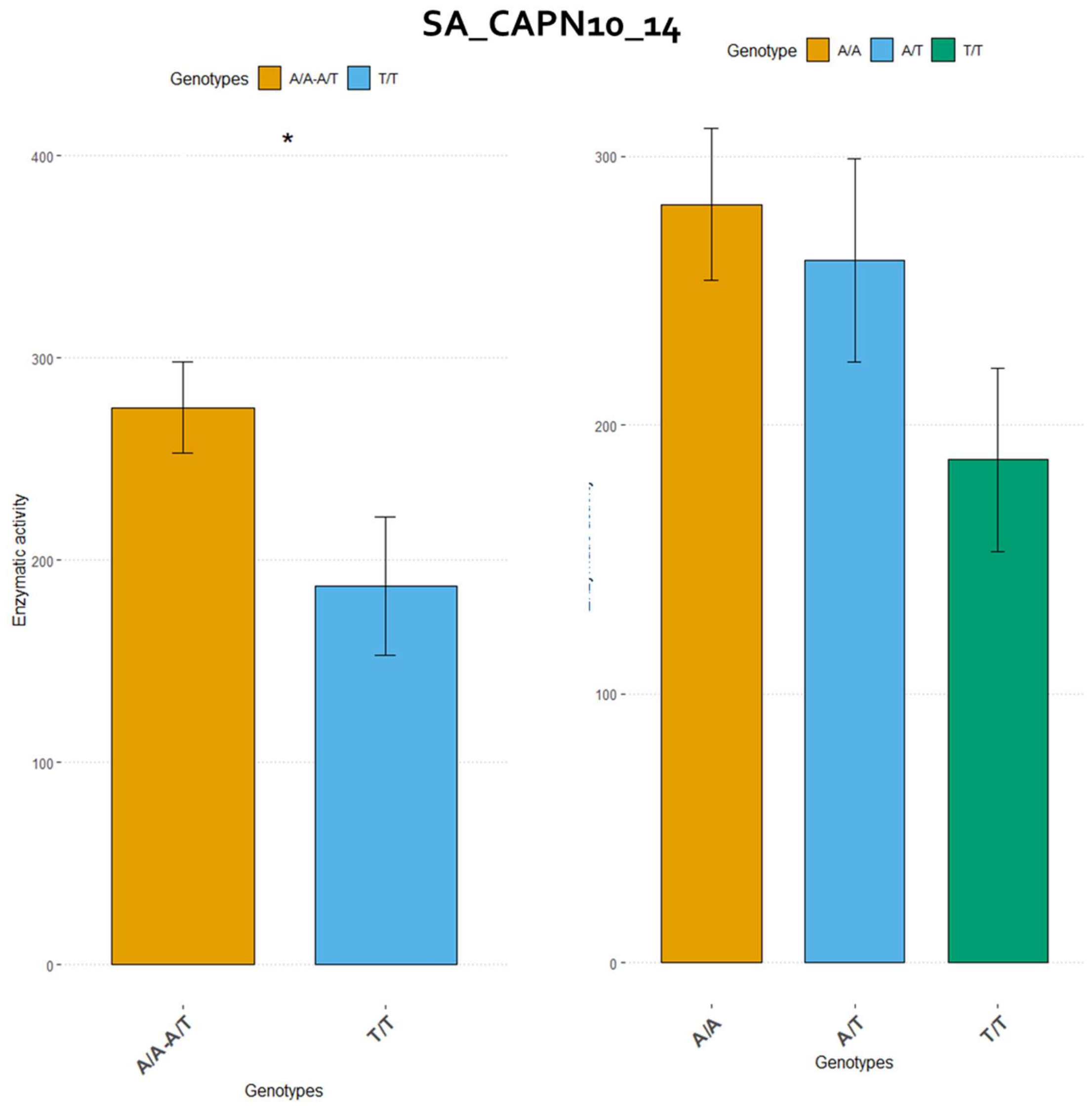


**Figure S2.** Enzymatic activity of Calpains by each genotype. SA_CAPN10_14 has a recessive model of inheritance.

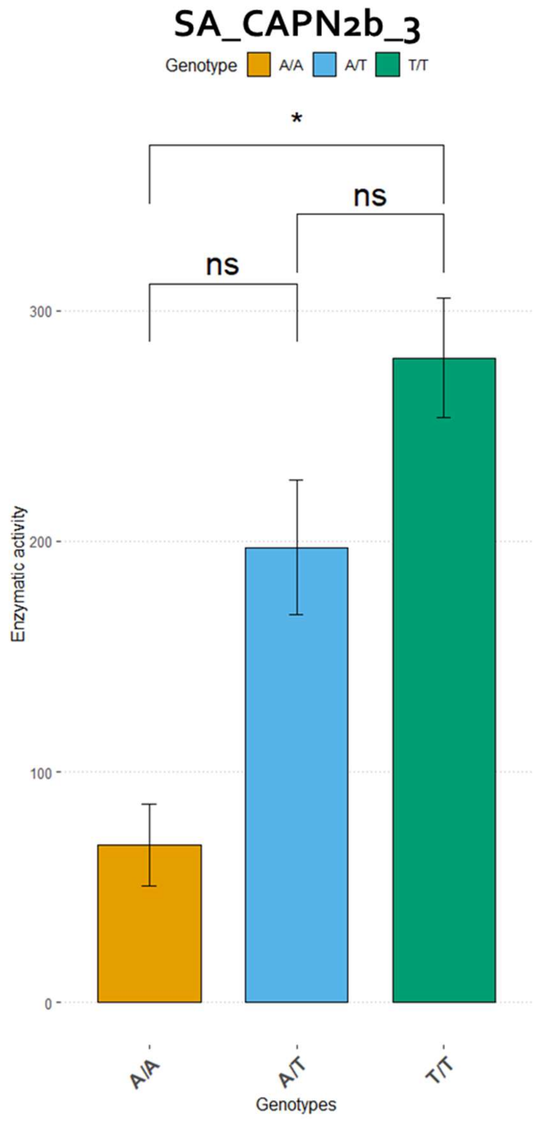


**Figure S3.** Enzymatic activity of Calpains by each genotype. SA_CAPN2b_3 has a log-additive model of inheritance.

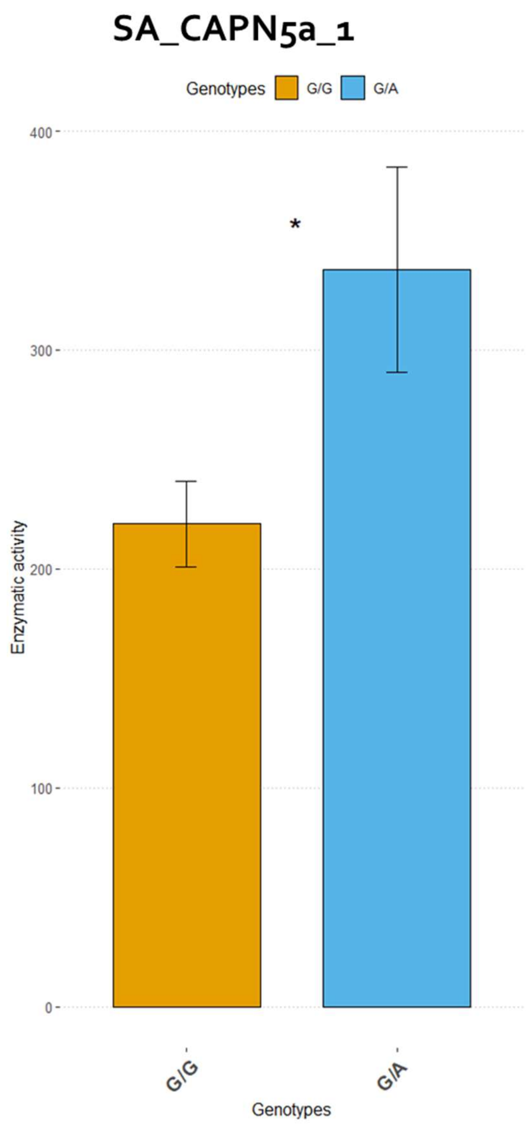


**Figure S4.** Enzymatic activity of Calpains by each genotype. SA_CAPN5a_1 lacks of the homozygous genotype AA.

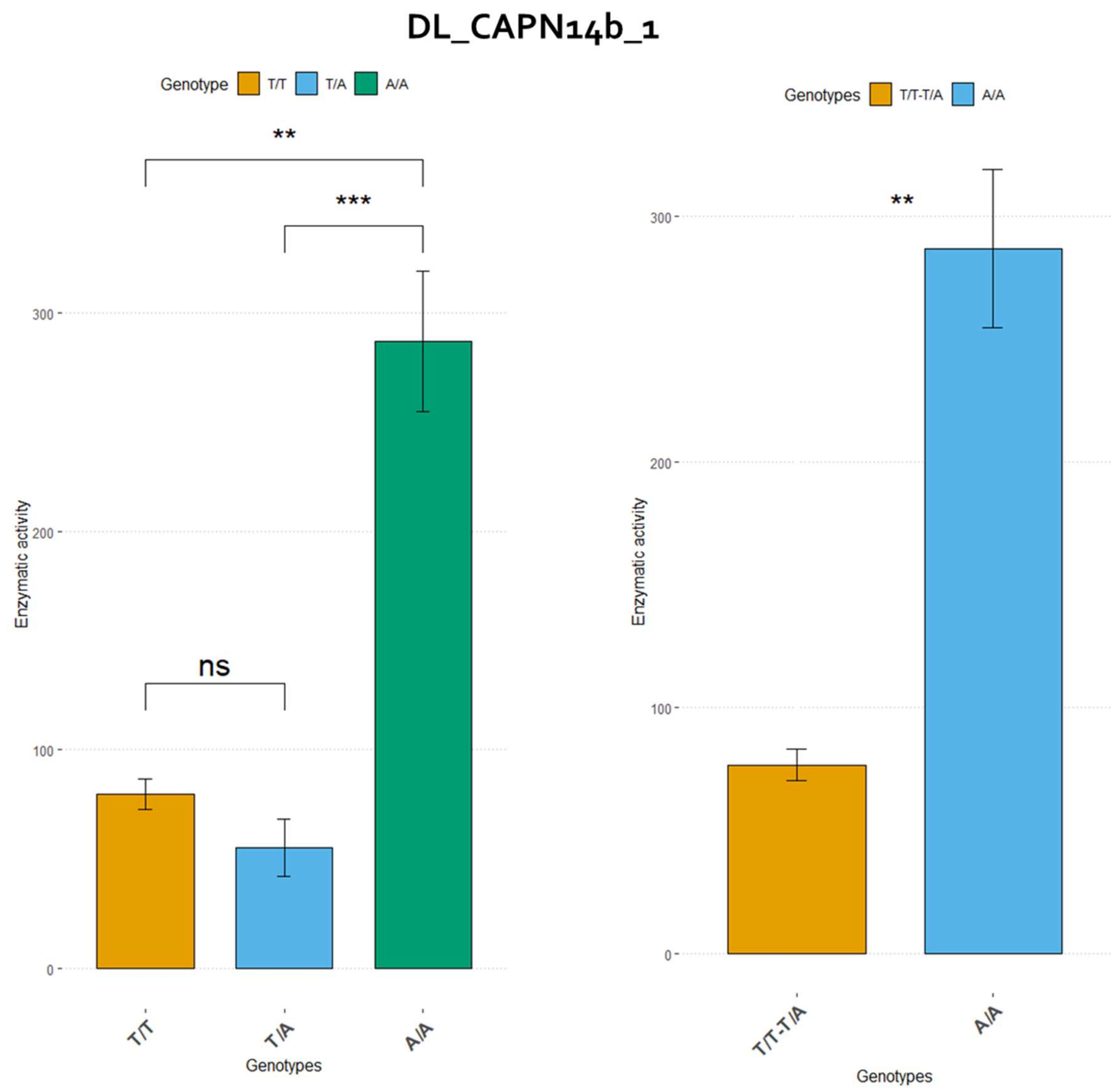


**Figure S5.** Enzymatic activity of Calpains by each genotype. DL_CAPN14b_1 has a recessive model of inheritance.

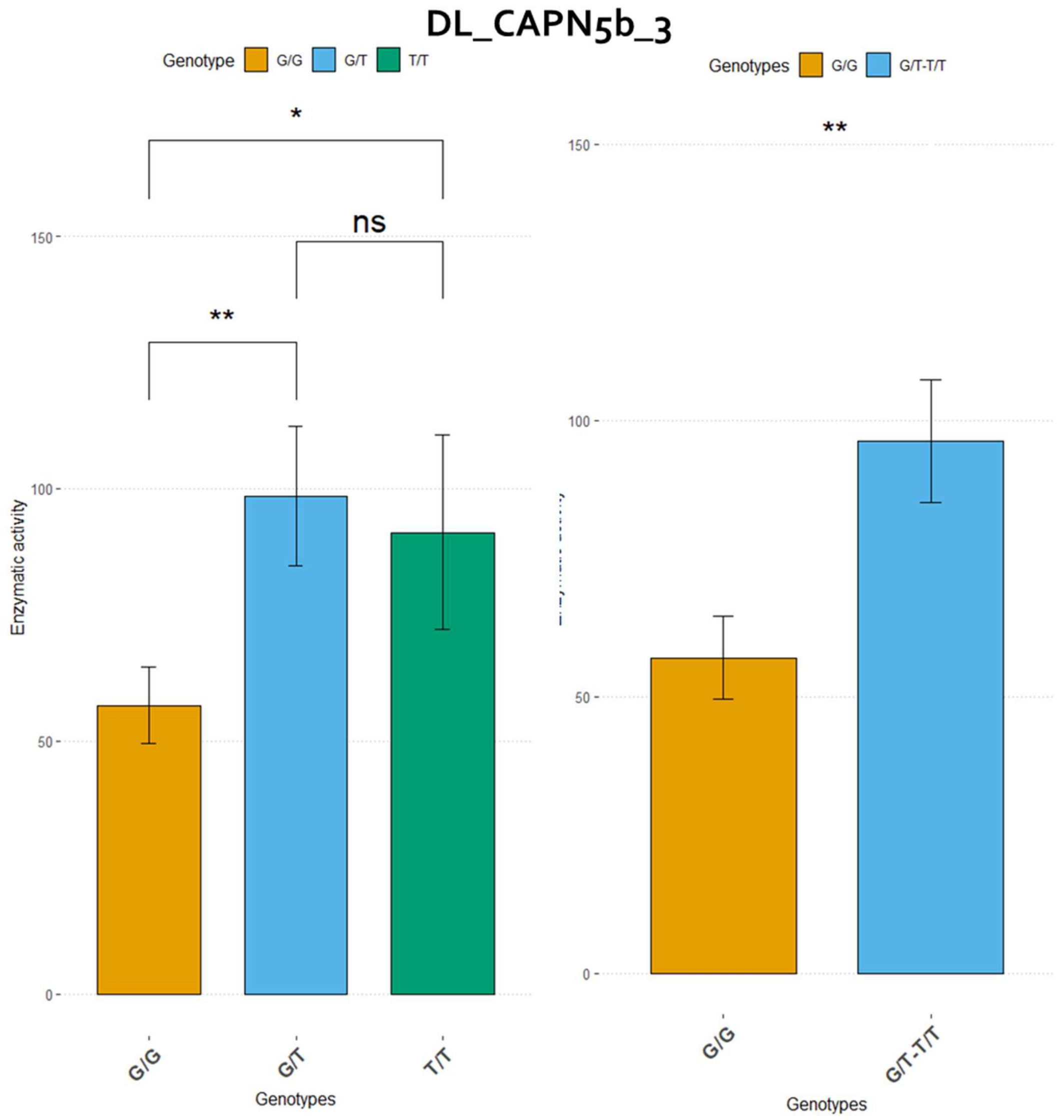


**Figure S6.** Enzymatic activity of Calpains by each genotype. DL_CAPN5b_3 has a dominant model of inheritance.

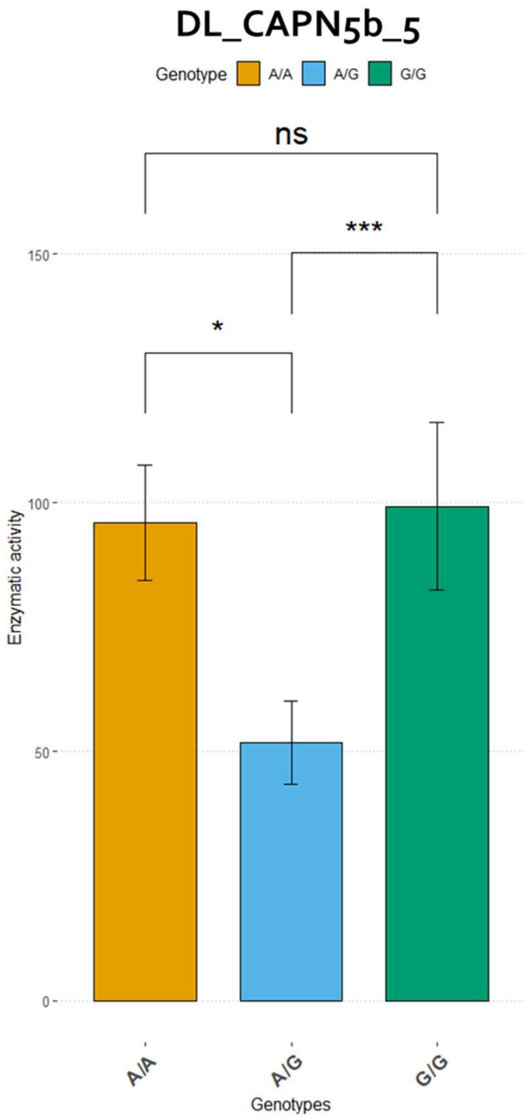


**Figure S7.** Enzymatic activity of Calpains by each genotype. DL_CAPN5b_5 has an overdominant model of inheritance.

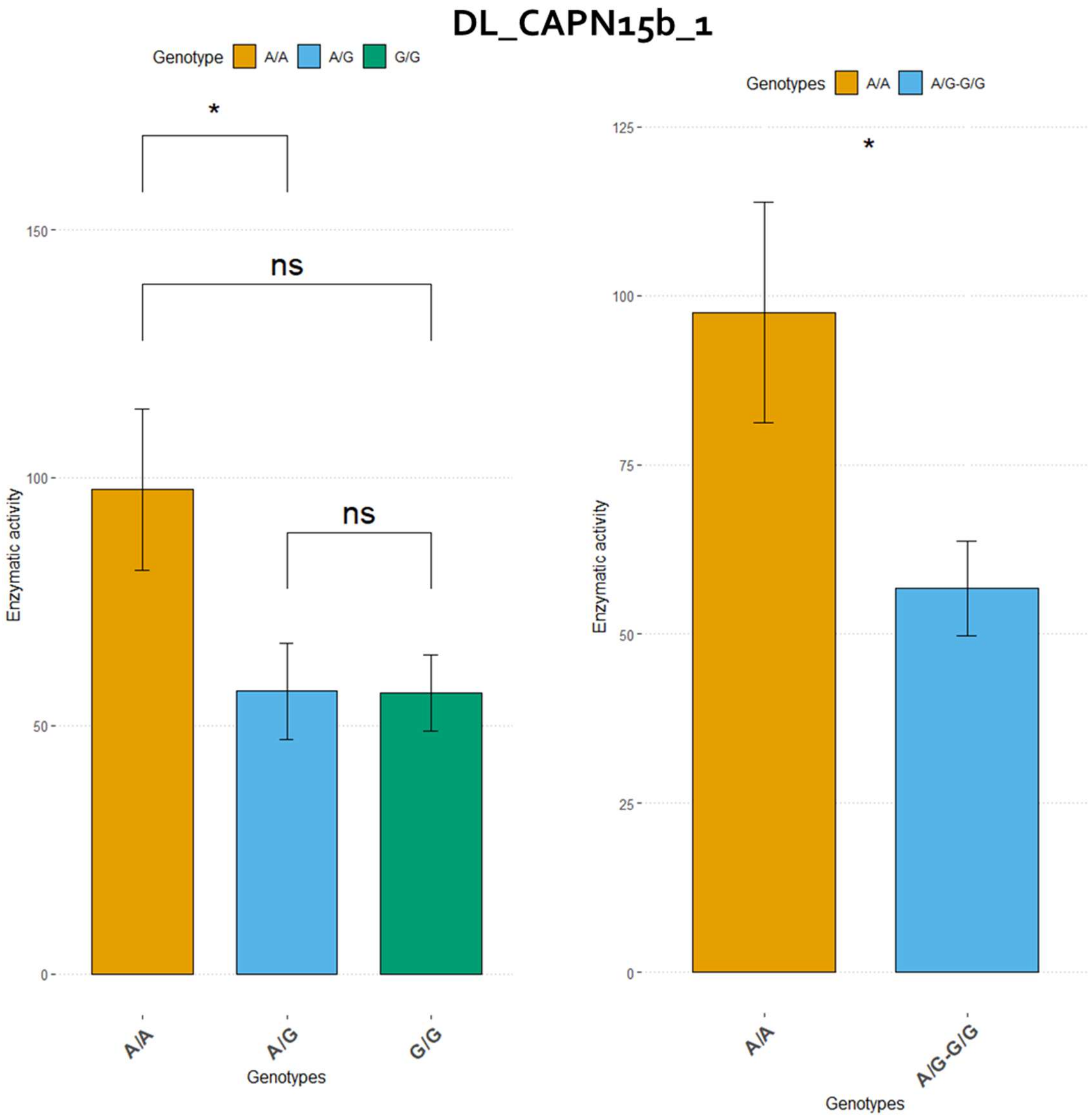


**Figure S8.** Enzymatic activity of Calpains by each genotype. DL_CAPN15b_1 has a dominant model of inheritance.

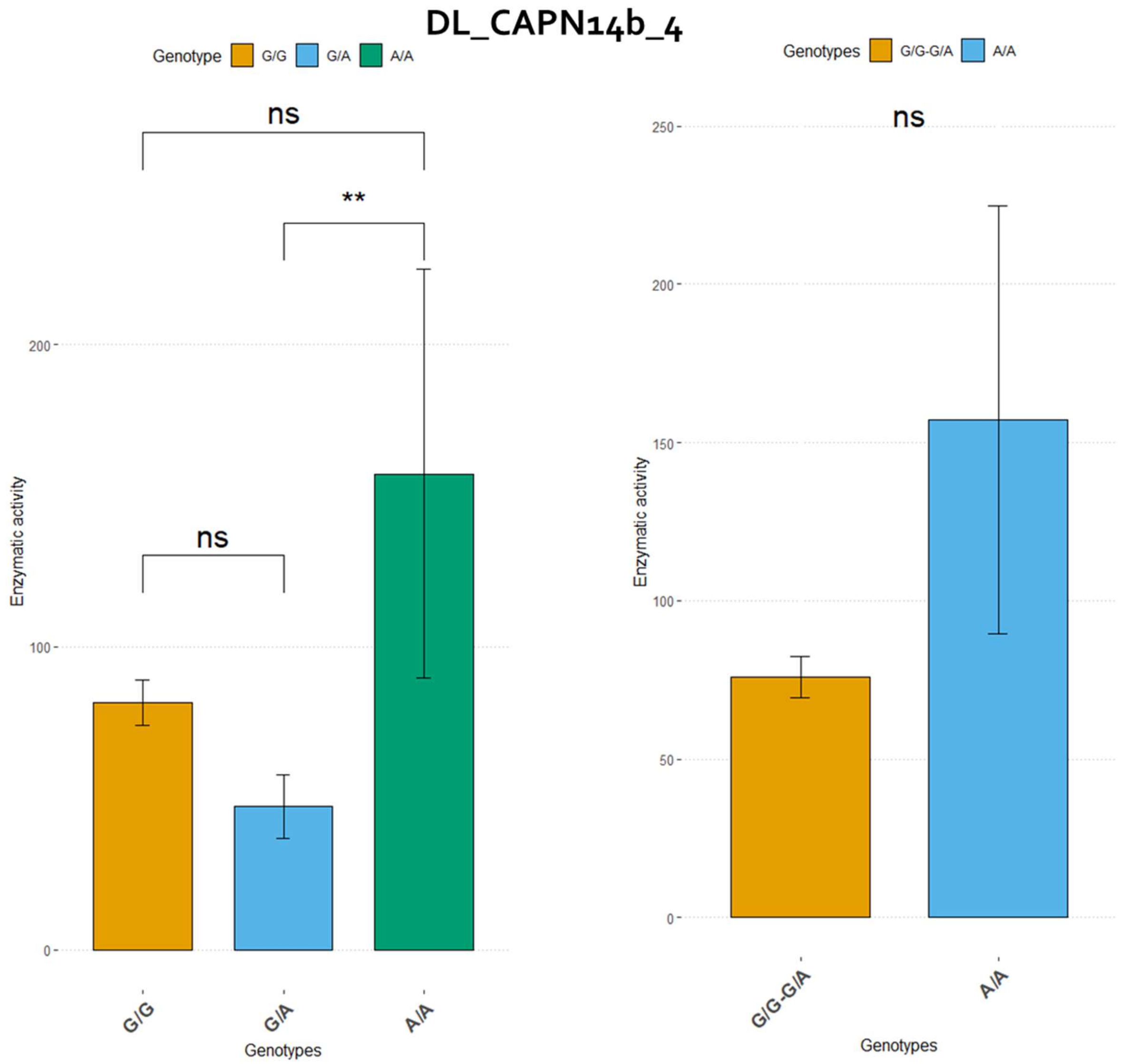


**Figure S9.** Enzymatic activity of Calpains by each genotype. DL_CAPN14b_4 has a recessive model of inheritance.

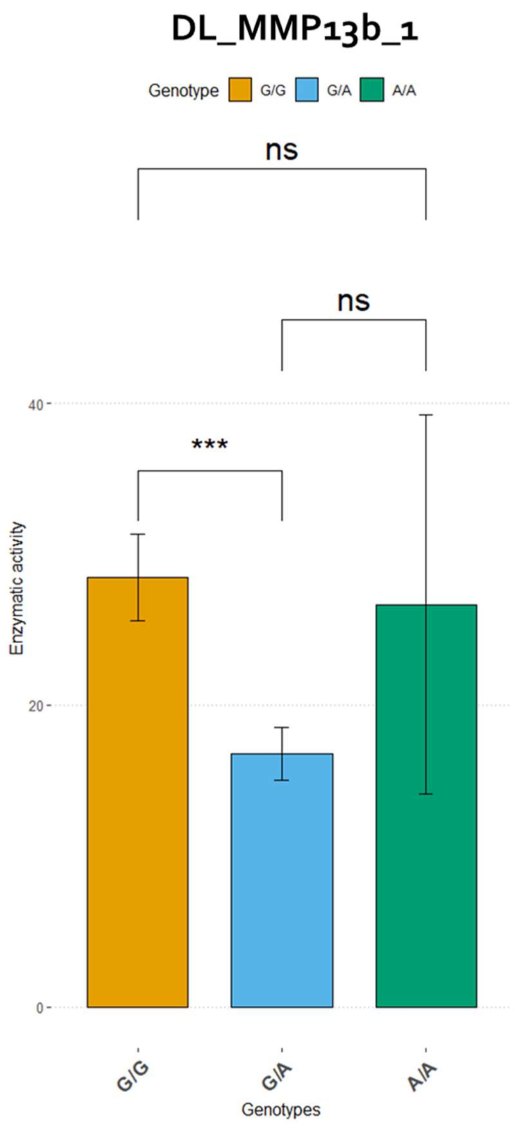


**Figure S10.** Enzymatic activity of collagenases (metalloprotease) by each genotype. DL_MMP13b_1 has an overdominant model of inheritance.

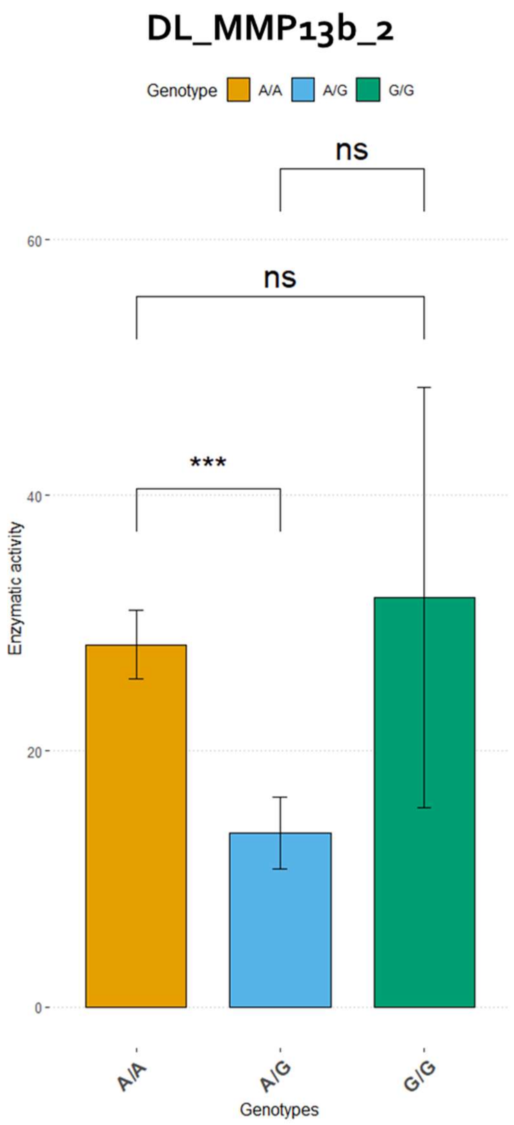


**Figure S11.** Enzymatic activity of collagenases (metalloprotease) by each genotype. DL_MMP13b_2 has an overdominant model of inheritance.
